# Cellular Prion Protein: From Physiology to Pathology

**DOI:** 10.3390/v4113109

**Published:** 2012-11-14

**Authors:** Sei-ichi Yusa, José B. Oliveira-Martins, Yoshiko Sugita-Konishi, Yutaka Kikuchi

**Affiliations:** 1 Division of Microbiology, National Institute of Health Sciences, Tokyo, Japan; Email: s-yusa@nihs.go.jp (S.I.Y.); kikuchi@nihs.go.jp (Y.K.); ykonishi@nihs.go.jp (Y.S.K.); 2 Current Address; Roche Diagnostics Deutschland GmbH, Mannheim, Germany; Email: jose.martins@roche.com

**Keywords:** neurodegenerative disease, prion, proteolytic cleavage, C1

## Abstract

The human cellular prion protein (PrP^C^) is a glycosylphosphatidylinositol (GPI) anchored membrane glycoprotein with two N-glycosylation sites at residues 181 and 197. This protein migrates in several bands by Western blot analysis (WB). Interestingly, PNGase F treatment of human brain homogenates prior to the WB, which is known to remove the N-glycosylations, unexpectedly gives rise to two dominant bands, which are now known as C-terminal (C1) and N-terminal (N1) fragments. This resembles the β-amyloid precursor protein (APP) in Alzheimer disease (AD), which can be physiologically processed by α-, β-, and γ-secretases. The processing of APP has been extensively studied, while the identity of the cellular proteases involved in the proteolysis of PrP^C^ and their possible role in prion biology has remained limited and controversial. Nevertheless, there is a strong correlation between the neurotoxicity caused by prion proteins and the blockade of their normal proteolysis. For example, expression of non-cleavable PrP^C^ mutants in transgenic mice generates neurotoxicity, even in the absence of infectious prions, suggesting that PrP^C^ proteolysis is physiologically and pathologically important. As many mouse models of prion diseases have recently been developed and the knowledge about the proteases responsible for the PrP^C^ proteolysis is accumulating, we examine the historical experimental evidence and highlight recent studies that shed new light on this issue.

## 1. Introduction

Transmissible spongiform encephalopathies (TSE diseases) or prion diseases are all fatal neurodegenerative conditions that include scrapie in sheep, bovine spongiform encephalopathy (BSE) in cattle, chronic wasting disease (CWD) in cervids, and kuru, Gerstmann-Sträussler-Scheinker syndrome (GSS), sporadic, familial, and variant forms of Creutzfeldt-Jakob disease (CJD) in humans. The central dogma of prion biology is that the normal cellular isoform of prion protein (PrP^C^) encoded by the highly conserved single-copy gene *Prnp* [1] is post-translationally refolded into a partially protease resistant and β-sheet-enriched conformation (generally termed PrP^Sc^) that is infectious [2]. However, neither the mechanism by which PrP^Sc^ causes neuronal dysfunction during prion disease nor the normal function of PrP^C^ is well defined. Although several functions have been suggested [3,4,5,6,7], none of these has been shown to be associated with prion disease pathogenesis or prion replication.

The neurotoxicity caused by prions is not simply explained by loss of a normal functional activity of PrP^C^, since mice lacking PrP^C^ are healthy and fail to develop prion disease [8,9]. Aguzzi’s group investigated whether prion pathology derived from neurotoxicity of PrP^Sc^, by grafting neural tissue overexpressing PrP^C^ into the brain of PrP^C^-deficient mice which are resistant to prions [10]. The grafted tissues kept producing high levels of PrP^Sc^ and induced localized infectivity after prion-inoculation in those mice, while the surrounding PrP^C^-deficient tissues showed no pathological changes and were not significantly damaged by PrP^Sc^. These results indicate that normal host prion protein is necessary for PrP^Sc^-induced neurotoxicity.

Additional studies addressed whether neurotoxicity and infectivity are two distinct phenomena. Collinge’s group developed double transgenic mice in which PrP^C^ is conditionally depleted in neurons after 9 weeks of age using the Cre-loxP system [9,11]. After prion inoculation of these mice, they initially suffered from development of clinical disease, but suddenly recovered from the disease and reversed spongiform neuropathology and behavioral abnormalities at around 9 weeks, even when brains of the mice contained high levels of PrP^Sc^. Furthermore, Chesebro’s group established transgenic mice expressing prion protein lacking the glycosylphosphatidylinositol (GPI) membrane anchor to prevent the prion proteins from localizing on the surface of cells [12]. These mice accumulated abnormal protease-resistant PrP^Sc^ in their brains after prion inoculation, and the brains were physically damaged by PrP^Sc^ amyloid plaques, but no clinical signs of disease were observed after 1 to 2 years compared to that seen in the non-Transgenic (Tg) control wild-type mice after 140 to 160 days. Thus, whatever the physiological function of PrP^C^, it has become increasingly clear that expression of PrP^C^ at the cell surface is necessary to mediate the full toxicity induced by PrP^Sc^. 

The uncoupling of neurotoxicity and infectivity is a growing concept in the prion field [13]. Collectively, PrP^Sc^ that is major constituent of prions can propagate using PrP^C^ as a substrate and then PrP^Sc^ may cause neuronal cell death using membrane bound PrP^C^ as a detonator. How this occurs is currently unknown, however.

## 2. Normal cellular prion protein, PrP^C^

Three Nobel Prizes have been awarded for seminal work performed in the prion field. Daniel Carleton Gajdusek received the award in 1976 for the discovery of “slow virus” that is now known as prion disease. Stanley B. Prusiner subsequently received the award in 1997 for the discovery of “prion” as the sheep scrapie agent and introduction of the “protein only hypothesis”, which stated that misfolded proteins are the infectious agents that cause prion diseases. Finally, Kurt Wüthrich received the award in 2002 for his contributions to the use of NMR to determine three-dimensional structures of proteins and nucleic acids in solution, including unraveling the structure of prion proteins using this technique.

According to the widely accepted “protein-only hypothesis” by Prusiner, proteinase resistant protein, PrP^Sc^, is infectious. Early studies by Weissmann’s group surprisingly demonstrated that PrP^Sc^ is an abnormal isoform of host-encoded cellular prion protein (PrP^C^) [1,14]. Since then, many scientific efforts have been performed to address the normal function of PrP^C^, but the suggested physiological functions of PrP^C^ do not completely explain all phenomena seen in prion diseases, and further research is needed. Several of these elements have been discussed in excellent recent reviews [15,16,17]. Here, we briefly elaborate on the processing of PrP^C^, involving its normal metabolism.

**Figure 1:**
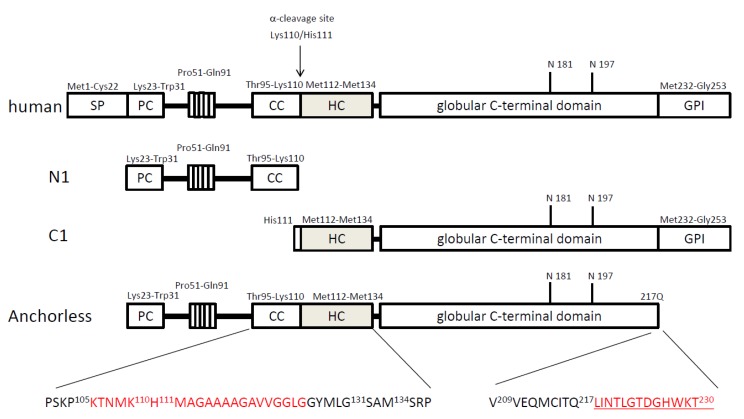
Diagrammatic representation of protein structure of normal cellular prion proteins. Based on the knowledge of physiological processing of prion protein (PrP^C^), it is likely that mature PrP^C^ (residues from 23 to 253), N-terminal (N1) (23 to 110), C-terminal (C1) (111 to 253), and anchorless PrP (23 to 230) may exist in normal brains of humans. Anchorless PrP seems to be unglycosylated and non-cleavable at putative α-cleavage site. The minimum sequence that causes spontaneous neurodegeneration when deleted in mice is shown in red. The additional open leading frame for anchorless PrP is underlined. SP, signal peptide; PC, positively charged region; CC, charged cluster region; HC, hydrophobic core region.

PrP^C^ is encoded on the short arm of human chromosome 20 (20q13) as 16 kb single copy PRNP gene and the open leading frame (ORF) is encoded on exon 2, which facilitates the cloning of the ORF by polymerase chain reaction (PCR) directly from genomic DNA. The human prion gene PRNP encodes a 253-residue precursor protein (see Figure 1). The first 22 N-terminal residues are post-translationally removed during transport to the cell surface. The last 23 C-terminal residues are excised after the addition of the glycosyl phosphatidylinositol (GPI) anchor. Thus, mature PrP^C^ consists of 208 residues. As PrP^C^ is variably glycosylated at two asparagine residues (N181 and N197), it exists as un-, mono- and di-glycosylated species. 

When Wilfried W. de Jong’s group compared PrP coding sequences in 26 mammalian species [18], they noted that a hydrophobic region in the middle of PrP^C^ is perfectly conserved, suggesting that the region is functionally important for PrP^C^. This hydrophobic core (HC) region is immediately adjacent to the putative cleavage site of PrP^C^, which is enriched in charged residues (Figure 1), designated the charged cluster (CC) region. The putative cleavage site has been commonly referred to as the α-cleavage site. 

Until more recently, the existence of the truncated form of PrP^C^ was not widely recognized by many prion biologists, since western blot analysis for brain homogenates or cell lysates showed several bands, which were attributed to differential glycosylation.

The discovery of the truncated form of PrP^C^ can be traced back to the early 1990s, when Prusiner’s group characterized PrP^C^ from Syrian hamster brain [19]. They found two PrP^C^ forms designated PrP^C^-I and PrP^C^-II which appeared to be generated from PrP^C^-I by limited proteolysis of the N-terminus, as confirmed by epitope mapping with different antibodies. Spontaneous degradation during the purification procedure was ruled out, since various protease inhibitors failed to alter the detection of the two protein forms. The same group further characterized PrP^C^-II and concluded that PrP^C^-II appears to lack the putative α-helical domain (codons 109-122), which is likely to be necessary for PrP^Sc^ formation. They also identified that chymotrypsin-like activity is a candidate mechanism by which a ~9 KDa fragment is proteolytically excised from the N-terminus of PrP^C^ [20].

In 1995, Gambetti’s group examined PrP^C^ metabolism in more detail. The group characterized the PrP forms present in normal and pathological human brains and in neuroblastoma cells [21]. They demonstrated that a COOH-terminal fragment of PrP^C^, which was named “C1”, is found in normal and CJD brains, as well as in human neuroblastoma cells. C1 turned out to have alternative N-termini starting at either His-111 or Met-112 using epitope mapping and radiosequencing. Thus, it seems that C1 contains at least the HC region (amino acids 112-133). Interestingly, both PrP^C^ and C1 seem to be anchored to the cell membrane, since both are mostly recovered in the membrane fraction and are released after treatment with phosphatidylinositol-specific phospholipase C (PIPLC) [21]. These data provide supporting evidence that C1 is not a degradation product generated during experimental procedures, as suggested by others [14,19].

In 1993, Harris’s group investigated chicken brain and murine neuroblastoma cells transfected with the chicken homologue of PrP^C^ [22]. Chicken PrP^C^ was demonstrated to undergo two cleavages as part of its normal metabolism. One product is a secreted form of full-length PrP^C^, which is released from the membrane when the GPI-anchor is cleaved. Importantly, they also found a second truncated form that accounts for most of the surface-anchored molecules in transfected murine cells, indicating that the machinery to generate C1 is highly conserved between species. The N-terminal cleavage fragment, termed N1, was found in medium from neuroblastoma cell cultures, as well as in chicken cerebrospinal fluid. We have also detected N1 fragments in the cell lysates of murine neuroblastoma N2a cells, although N1 is unstable even when stored at -80 ˚C in the presence of proteinase inhibitors, resulting in analysis having to be performed immediately to avoid degradation (unpublished data). Collectively, PrP^C^ appears to have two physiological forms on cell surfaces; one is full-length and the other is the truncated C1 form, and both may have functions (Figure 1). Whether the secreted forms (GPI-cleaved PrP or N1) have physiological functions will require more detailed investigation in the future. 

Interestingly, physiological anchorless prion protein may exist in humans due to alternative splicing of exon 2 of the PrP gene, which eliminates the GPI-anchor [23] (see Figure 1). Moreover, analysis of the transcriptome derived from zebrafish embryo mRNA present at 5.3 hours postfertilization (hpf) indicates splice variants of PrP-2 which lacks GPI-anchor [24]. Since the anchorless form is unglycosylated and unprocessed, the full-length anchorless PrP^C^ may have physiological function in the absence of N-linked glycosylation and without α-cleavage [23]. Transgenic mice expressing artificial GPI-anchorless PrP^C^ protein also showed that the exogenous anchorless PrP^C^ is unglycosylated, reside in the cytosol, and is secreted [12]. However, the glycosylation status is controversial, since GPI-anchorless PrP^C^ is fully glycosylated in different cell types [25]. Collectively, there are at least four forms of normal cellular prion protein; full-length GPI-anchored, N1, C1, and GPI-anchorless (Figure 1). None of the physiological functions of all these prion proteins has so far been clarified.

## 3. Transgenic mouse models

**Figure 2:**
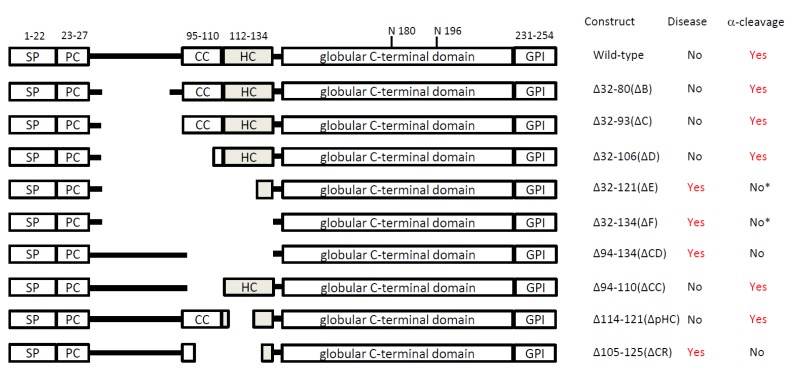
Schematic of PrP constructs and comparison of α-cleavage. All constructs shown here are derived from murine PrP^C^. The constructs were inserted into half genomic expression vectors, containing the promoter, 5’intronic, and 3’untranslated sequences of the murine prion protein gene [31,32]. All mice are *Prnp*^0/0^ background. Spontaneous neurodegeneration (Yes), C1 generation (Yes) are shown, according to the publications. *The sizes of C1 and exogenous PrP are similar so that it might be difficult to judge.

Prion diseases were originally thought to be caused by a kind of slow-virus, as suggested by Gajdusek. Subsequently, the nature of materials that cause prion diseases was hypothesized by Prusiner to be the PrP^Sc^ protein, but not a virus. However, a new paradigm has appeared since normal cellular PrP^C^ was found and examined. In 1998, Weissmann’s group produced several truncated forms of PrP^C^ to identify the regions necessary for its pathogenic conversion, since PrP^C^-deficient mice are resistant to scrapie which is induced by mouse-adapted sheep prions [26]. Unexpectedly, they found that PrP lacking residues 32-121 or 32-134 caused severe ataxia and neuronal death limited to the granular layer of the cerebellum as early as 1-3 months after birth (Figure 2). Importantly, attempts to transmit disease with brains of these spontaneously ill mice failed, no PrP aggregation was found, and the introduction of a single copy of the wild-type *Prnp* allele (PrP^C^) rescued the phenotypes. Thus, PrP lacking residues 32-121 or 32-134 (ΔF) causes neuronal dysfunction irrespective of “infectious prions”. Before this report, in 1990 Prusiner’s group produced transgenic mice that have a leucine substitution at codon 101 (corresponding to P102L in human), which is the genetic basis of the human inherited prion disease, Gerstmann-Sträussler-Scheinker syndrome [27]. The mice also spontaneously developed prion diseases, showing spongiform degeneration, astrogliosis, amyloid plaques, and infectivity to other mice [28,29]. Based upon these reports, we have categorized the transgenic mouse models in two groups; one with the non-infectious “ΔF phenotype”, the other with the infectious “PrP^Sc^ phenotype” (Table 1. Due to limited information some transgenic mice are excluded from these categories.

Weissmann’s group produced mice expressing PrP with several amino-proximal deletions in addition to ΔF (32-134) [26]. Some of these were normal, while others suffered from spontaneous neurological diseases which were non-infectious and did not generate protein aggregates. Interestingly, there was a strong correlation between the neurotoxicity caused by the truncated forms of PrP and the blockade of their proteolysis (see Figure 2). Wild-type PrP^C^ (non-toxic), exogenous PrP^C^ (non-toxic) (i.e. *Tga*20), ΔB(32-80)(non-toxic), ΔC(32-93)(non-toxic), and ΔD(32-106)(non-toxic) all showed the appearance of C1 by western blot analysis and no cerebellar syndrome (Figure 2). On the other hand, ΔE(32-121) (toxic) and ΔF(32-134) (toxic) did not show detectable C1 and caused neurodegenerative symptoms, (however in the publication of the ΔF mice it is difficult to judge C1 due to their similar size of ΔF(32-134) and C1). The absence of C1 in ΔF(32-134) is also examined in an *in vitro* assay using the HpL 3-4 *Prnp*-deficient cell line [30], indicating that ΔF is slightly larger than C1. The different phenotypes between the former (non-toxic) and the latter (toxic) are not simply explained by aberrant cellular localization of the truncated forms or by distinct expression levels, since all forms were shown to be expressed on the cell surface and the levels of transgene expression were comparable among the mice [26].

**Table 1:** Transgenic mouse models with mutant prion proteins.

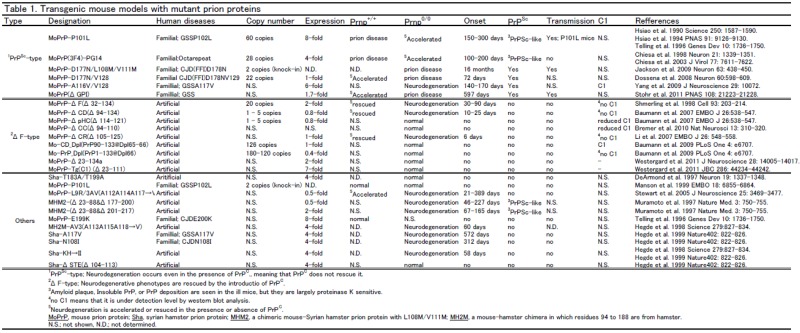

Aguzzi’s group developed transgenic mice, expressing PrP variants lacking residues 94-134(ΔCD) or 114-121(ΔpHC) (Figure 2) [33]. These deletion mutants are quite interesting because 94-134 encompasses the CC, α-cleavage and HC sites, and 114-121 includes the N-terminal palindrome sequence (AGAAAAGA), which is highly conserved among species and is thought to be important for the conversion into PrP^Sc^ [34,35]. These transgenic mice showed distinct phenotypes on a *Prnp*^0/0^ background. ΔCD (94-134) mice spontaneously developed neurodegenerative diseases, while ΔpHC (114-121) mice did not [33]. The striking difference compared to ΔF (32-134) mice is that ΔCD (94-134) mice show more severe neurodegeneration, since the phenotypes were observed even on a *Prnp*^+/+^ background. To overcome the neurotoxicity caused by ΔCD (94-134), either low expression of the exogenous ΔCD (94-134) or the introduction of exogenous high PrP^C^ expression, such as in *Tga*20 mice, are required [33]. Upon PNGase-F treatment of the brain homogenates from those mice, PrP^C^ displayed a significant amount of C1, whereas C1 formation was reduced in ΔpHC (114-121) and absent from ΔCD (94-134) [33], supporting the model dictating that blockade of α-cleavage of PrP^C^ makes it toxic. Similar results were also obtained in Tg(ΔCR) mice by Harris’s group where PrP^C^ was deleted between residues 105 and 125, which include the CC and HC regions (105-125). This deletion appears to be the minimum mutation required to block α-cleavage of PrP^C^ *in vivo* using transgenic mice. Interestingly, accumulating results have shown that PrP peptide 106-126 is highly toxic in both murine and human neurons. However, further investigation is required to clarify the relation between PrP 106-126 and α-cleavage phenomenon. The same group also showed that ΔCR (105-125) is expressed on the surface of neurons, but C1 generation is undetectable by western blot analysis of brain homogenate (Figure 2) [36].

All of the above mice had spontaneous neurodegenerative dysfunction and importantly the dysfunction can be rescued by normal PrP^C^ in a dose dependent manner of PrP^C ^expression. In contrast, other transgenic mice including MoPrP-P101L, MoPrP-PG14, MoPrP-D177N and MoPrP-A116V showed spontaneous neurodegeneration, but the phenotypes were not rescued by PrP^C^ expression, which instead exacerbated the phenotypes (Table 1) [27,28,29,37,38,39,40,41]. Most strikingly, these mice appear to spontaneously generate PrP^Sc^ or amyloid plaque. In addition, their disease is often transmissible if “species barrier” is considered. Thus, these transgenic mice are likely a valuable model for prion disease. Although the generation of C1 was not assessed in these mice, α-cleavage of these mutant PrPs would occur fully or partially, since an *in vitro* system by Oliveira-Martins et al. showed that α-cleavage is unexpectedly tolerant to mutations unless a large portion of PrP^C^ is deleted [30]. However, it still remains to be determined how “PrP^Sc^-type” could be explained by the “α-cleavage phenomenon”.

In 1995 Gambetti’s group described that an additional fragment longer than C1, designated C2, is present in substantial amounts in CJD brains, but not in normal controls [21]. Thus, C1 and C2 seem to be completely different in terms of physiological function, because C2 appears to be pathogenic. Interestingly, C2 seems to have the 3F4 epitope, meaning that C2 contains at least the residues between 90 and 104, which is upstream of the α-cleavage site (110 or 111). Thus, C2 is the fragment of PrP^C^ that was not cleaved at the α-cleavage site. 

 In 2006, Telling’s group produced transgenic mice, expressing GFP-tagged murine PrP at codon 22 [42]. In addition to the study of the GFP-PrP^C^, they tested a murine adopted scrapie (RML) infection. Interestingly, they used both PNGase F and Proteinase K to detect PrP^Sc^. Normal mice showed two bands on WB, PrP^C^ and C1, and both bands were completely digested by Proteinase K treatment at 20 μg/ml. On the other hand, normal mice infected with RML showed three bands, PrP^C^, C1 and C2. The data demonstrates that after Proteinase K treatment at 20 μg/mL C2 is retained, but not PrP^C^ and C1, suggesting that C2 is the PrP^Sc^ [43].

As previously described, Prusiner’s group produced Tg mice overexpressing MoPrP-P101L, which spontaneously developed central nervous system dysfunction and transmissible, even though Proteinase K resistant PrP^Sc^ was not detected. In 2005, Telling’s group revisited this issue [44]. They used the cold PK technique (Proteinase K treatment on ice), PNGase F, and PrP^Sc^-specific antibody (15B3) to show the appearance of a proteinase resistant form of MoPrP-P101L with molecular weights corresponding to C2. Thus, PNGase F can be a powerful tool to detect proteinase K sensitive PrP^Sc^, because contrary to Proteinase K treatment, which can completely digest the target protein depending on incubation time and concentration, the experimental impact of varying PNGase F treatment conditions is minimal. It will be interesting to test for the presence of such PrP^Sc^ using a similar technique in the MoPrP-PG14, MoPrP-D177N, and MoPrP-A116V transgenic mice.

STE(104-113), G123P, KH→II(K110H111 to I110I111 substitutions), AV3(V113V115V118 to A113A115A118 substitutions), A117V (human GSS) and N108I (human CJD) on hamster PrP^C^ [45,46]. Among these mice, KH→II, A117V, AV3, and N108I PrP expression induced spontaneous neurodegeneration, but importantly, PrP^Sc^ was not detected in these mice. Previously, the same group studied PrP translocation at the endoplasmic reticular (ER) membrane and revealed unusual features in its biogenesis. They found more than one topologic form of full-length PrP^C^ synthesized in cell-free translation systems, including at least secPrP, NtmPrP, and CtmPrP [45]. Interestingly, they also showed that favored synthesis in the CtmPrP form strongly correlates with neurodegenerative phenotypes such that mice expressing KH→II (toxic), A117V (toxic), and AV3 (toxic) have relatively more CtmPrP in their brains. They concluded that expression of CtmPrP produces neurodegenerative changes. It remains to be determined whether the neurodegenerative phenotypes by KH→II, A117V, and AV3 mice are rescued by PrP^C^ or whether C1 is generated in these mice. 

**Figure 3:**
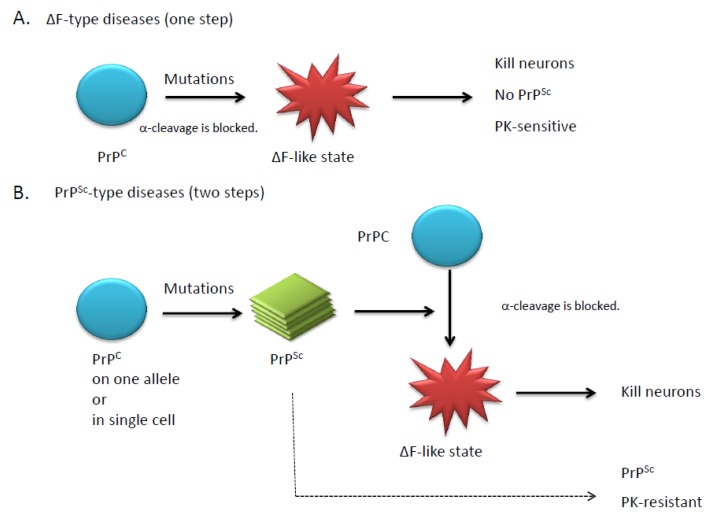
(**A)** When PrP^C^ is not cleaved by deletion or mutation, the PrPs elicit neurotoxicity. **(B)** When the α-cleavage of PrP^C^ is blocked, the unstable PrPs tend to spontaneously aggregate to generate PrP^Sc^. The generated PrP^Sc^ then affect the α-cleavage of neighboring PrP^C^ to elicit neurotoxicity or the conversion process from PrP^C^ to PrP^Sc^ by itself transduces a toxic signal. A mutation or deletion may occur on single allele or in single cell.

 Mice expressing the “ΔF type” PrP have so far not been shown to generate a relevant C2 fragment. On the contrary, it is possible that all mice with the “PrP^Sc^ type” may show a prominent C2. The finding that PrP^Sc^ might be the C2 is interesting, because PrP^Sc^ is not cleaved at the α-cleavage site. Therefore, expression of “PrP^Sc^ type” mutants would also affect α-cleavage because certain mutations of PrP^C^ result in the generation of PrP^Sc^ that is resistant to α-cleavage or PrP^Sc^ that blocks α-cleavage of PrP^C^ through interactions with each other (see Figure 3).

**Figure 4:**
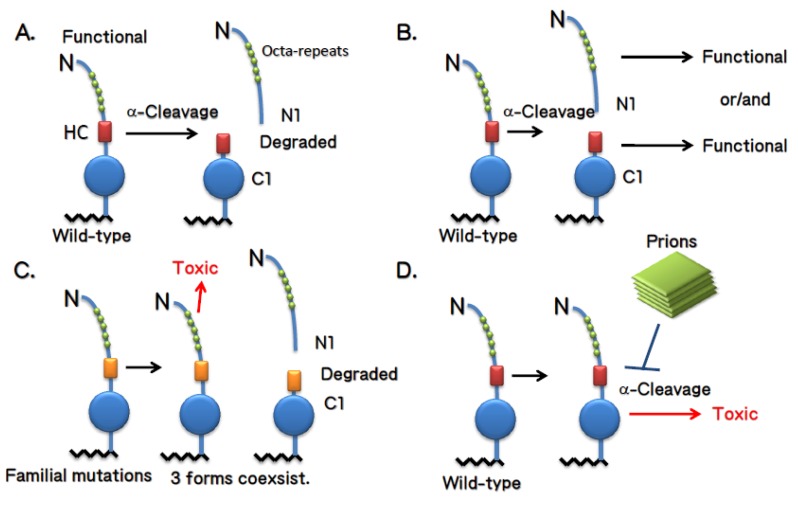
**(A)** Full-length PrP^C^ has a function so that it is immediately cleaved at α-cleavage site after activation. **(B)** Either N1 or C1 is an active form so that it is cleaved at α-cleavage site to activate it. **(C)** PrP^C^ with mutations is not completely cleaved so that the uncleavable PrP show some residual toxicity. **(D)** Spontaneously generated PrP^Sc^ on one gene or derived from single cell blocks the α-cleavage of the remaining PrP^C^, resulting in neurodegeneration. Infectious Prions also blocks the α-cleavage of host PrP^C^.

Is the reduction of C1 or inefficient α-cleavage of PrP toxic? One hint to answer this question can be deduced from the experiments performed by Aguzzi’s group [33]. As discussed above, C1 formation was reduced in mice expressing ΔpHC (114-121) and absent from when ΔCD (32-134) is expressed (Figure 2). It might be possible that the presence of ΔpHC still retains un-cut PrP^C^, which was supposed to be cleaved at the correct position and time. Therefore, presence of ΔpHC may retain some residual toxicity, which cannot be observed in the short life span of a mouse. Accordingly, the introduction of ΔpHC into ΔCD mice enhanced the phenotypes [33], suggesting that ΔpHC induces residual toxicity. In addition, the introduction of ΔpHC into ΔF mice partially rescued the ΔF phenotypes [33], indicating that ΔpHC also retains a function of PrP^C^, since it is partially cleaved. These findings support a mechanistic model that cleaved and un-cleavable PrPs exist simultaneously with ΔpHC; one is non-toxic and able to rescue ΔF phenotypes and the other is toxic and enhances ΔCD neurotoxicity (Figure 4C).

What is the nature of neurotoxicity caused by expression of PrPs of the “ΔF type”? Although the endogenous physiological C1 does not contain residues 1 to 110 or 111, Harris’s group pointed out that the artificial PrP constructs, such as Δ32-134, Δ94-134, and Δ105-125, retain the N-terminal 9 amino acids (residues 23-31, KKRPKPGGW) (Figure 2). Therefore, they engineered transgenic mice expressing a PrP mutant with 23-134 deleted (Δ23-134) [47]. According to their report, these mice surprisingly did not display clinical symptoms or neuropathology, in contrast to mice expressing Δ32-134, Δ94-134, or Δ105-125 [26,33,48]. Importantly, Δ23-134 (non-toxic), Δ32-134(toxic), Δ94-134(toxic), andΔ105-125(toxic) PrPs are expressed on the surface of cells, but did not seem to be cleaved to generate C1, thereby rejecting the notion that the blockade of α-cleavage correlates with neurotoxicity by PrP mutants. In addition, neither Δ23-134 (non-toxic) nor Δ32-134 (toxic) PrPs were efficiently endocytosed, while Δ105-125 (toxic) PrP was efficiently endocytosed in an *in vitro* cell culture system, indicating that the lack of toxicity of the Δ23-134 mutant cannot be attributed to its impaired endocytosis. This report suggests that the toxicity caused by PrP mutants that cannot generate C1 relates to presence of the nine amino acids (KKRPKPGGW). Similar evidence was previously shown by Prusiner’s group, where a Δ23-88 mutant PrP was introduced into *Prnp*-deficient mice [49]. Their transgenic mice seemed to be normal, but the 23-88 mutant PrP does not seem to be functional, because it failed to rescue the mice from the Purkinje cell death in Ngsk *Prnp*^0/0^ mice [50]. Thus, both Δ23-134 (un-cleavable) and Δ23-88 (cleavable) mutant PrPs might be non-functional. 

As discussed in the Introduction, both function and toxicity require prion protein localization at the plasma membrane. Consistent with this requirement, PrPΔCDs lacking residues 94-134 or the GPI-anchor were shown to be non-toxic [33]. In addition, expression of GPI-anchorless PrP did not rescue Mo-PrPΔF(Δ23-134), although it did show partial residual rescue [51]. Recently, Prusiner’s group produced Tg(PrP,ΔGPI)-expressing mice on a *Prnp*^0/0^ background [52]. Mice engineered with low expression of the exogenous ΔGPI mutant remained healthy, as previously reported [12]. In contrast, 1.7-fold higher expression of the ΔGPI mutant compared with PrP^C^ expression levels of wild-type mice resulted in late-onset, spontaneous neurologic illness accompanied by amyloid plaques. In addition, the disease was transmissible. Thus, Tg(PrP,ΔGPI) mice can be categorized into the “PrP^Sc^ type”. Because anchorless PrPs are not cleaved, it might be possible that the un-cut PrP tends to spontaneously aggregate (Figure 3B).

In conclusion, there have been at least two major types of transgenic mouse models produced to date. The first type directly produce PrP^Sc^ derived from exogenous mutant PrP transgenes so that the mice suffer from prion disease and the disease can be transmissible to other mice. Thus, endogenous PrP^C^ expression rather enhances the disease in these mice. The second type has nothing to do with prion disease in terms of PrP^Sc^ and transmissibility. These mice show neurodegenerative phenotypes in the absence of PrP^C^. Thus, the introduction of PrP^C^ rescues the phenotypes of these mice. In these mice, the exogenous artificial PrP mutants are extremely resistant to “α-cleavage”, while PrP^C^ is mainly cut into two fragments, N1 and C1.

How could the divergent phenotypes of these two mouse models be explained by a unified hypothesis? One hypothetical model to explain this dichotomy is illustrated in Figure 3. We propose that when PrP^C^ is not cleaved at all by deletion or mutation, the PrPs elicit neurotoxicity (ΔF type) or the PrPs spontaneously aggregate to generate PrP^Sc^ (PrP^Sc^ type), depending upon the mutations involved. When PrP^C^ is partially cleaved by mutation, three forms should exist simultaneously: un-cut PrP, N1, and C1 (i.e. ΔpHC). Thus, un-cut PrP may induce mild neurotoxicity that cannot be observed under normal conditions. We further propose that neurotoxicity by the un-cut PrP is evident in the presence of other mutant PrPs, since double transgenic mice with ΔpHC (non-toxic) and ΔCD (toxic) enhanced the neurodegenerative phenotypes of ΔCD mice [33]. On the other hand, expression of ΔpHC can partially rescue the neurotoxicity induced by ΔF (toxic). It might be possible that N1 or C1 derived from ΔpHC rescues the neurotoxicity induced by ΔF. Furthermore, we predict that ΔCD expression cannot be rescued by ΔpHC because ΔCD possesses a long N-terminal domain, which might block the functions of N1, C1, or full-length PrP^C^.

## 4. Doppel and Shadoo

### 4.1. Doppel

In order to address the function of PrP^C^, several PrP^C^-deficient mouse lines were developed by many laboratories, including Zrch I, Rcm0, Npu/Edbg, Ngsk, Rikn, and Zrch II [8,53,54,55,56,57,58]. Zrch I and Npu mice developed and reproduced normally, while Rcm0, Ngsk, Rikn and Zrch II mice exhibited severe ataxia and Purkinje cell loss in later life, as well as demyelination of peripheral nerves. Spontaneous cerebellar neurodegeneration seen in these mice led to the discovery of doppel (Dpl), a protein structurally related to PrP^C^ [54]. The mouse gene *Prnd* (Dpl) is located 16 kilobases downstream of *Prnp* (PrP^C^) and encodes a 179-amino acid protein. Dpl is absent from the brain and is highly expressed in testis under normal physiological condition. In accordance with its expression, Dpl-deficient mice showed a male infertility phenotype [59,60]. The deletions designed in Rcm0, Ngsk, and Zrch II mutants resulted in the generation of high levels of Dpl expression in the brain by an unusual mechanism involving exon-skipping and intergenic splicing. Interestingly, the introduction of a single wild-type PrP allele fully rescued the deleterious phenotype caused by the ectopic expression of Dpl in the brain [58], indicating that Dpl has a functional overlap with PrP^C^. Dpl shows structural homology with the C-terminal globular domain of PrP^C^, but lacks the N-terminal octarepeats and the hydrophobic core (HC) [61], suggesting that Dpl is not cleaved at the same position as PrP^C^. As expected, murine Dpl appears as a single band of ~17 KDa after PNGase F treatment [62], although human Dpl from testis additionally possesses an O-bound oligosaccharide [63,64]. Interestingly, Aguzzi’s group produced mice expressing fusion proteins in which the CC and HC regions of PrP^C^ (residues 90-133) (cleavable) were inserted into the middle of Dpl (between 65 and 66), and the chimeric CD-Dpl proteins are cleaved into two fragments [65]. These mice did not show the neurodegeneration phenotype seen by ectopic Dpl expression in several PrP^C^-deficient mice (i.e. Rcm0, Ngsk, Rikn and Zrch II), although these fusion proteins are artificial so their functional integrity is unclear. However, the CD-Dpl expression in either PrP-ΔF (Δ32-134) or PrP-ΔCD (Δ94-134) mice partially rescued the severe neurodegeneration caused by expression ΔF or ΔCD [65], indicating that the CD-Dpl proteins are at least partially functional. The same group also produced mice expressing the PrP-Dpl proteins in which the N-terminal domain of PrP (1-133) is fused to the C-terminal domain of Dpl (66-179). These PrP-Dpl fusion proteins are not toxic in the PrP^C^-deficient mice, even in the absence of C1-like molecules as shown by western blot analysis, implying no correlation between toxicity and lack of C1. It is possible that the PrP-Dpl fusion did not cause neurodegeneration because it is non-functional, but this does not seem to be the case as it partially, albeit weakly, rescued the ΔF and ΔCD phenotypes [65]. One possible explanation for the rescue of the ΔF and ΔCD phenotypes by PrP-Dpl is that the fusion protein still retains capacity to generate a residual level of C1 fragment that is below detection level by normal western blot analysis, since weak C1 expression was shown to partially rescue theΔF phenotypes in ΔpHC and ΔF double transgenic mice [33]. In addition to the α-cleavage phenomenon, one could conclude from these studies that the N-terminal 9 amino acids (23-31) and residues 90 to 133, which include the CC, α-cleavage site, and HC domains, but not the octarepeats, are sufficient to rescue the toxicity caused byΔF, ΔCD, and Dpl.

### 4.2. Shadoo

Database analysis by Gready’s group identified a novel gene named SPRN (Shadoo), shadow of prion protein (Sho) [66]. Dpl resembles the α-helical C-terminal half of PrP^C^, while Sho is reminiscent of the flexible N-terminal half, although Sho is extracellular and GPI-anchored. The SPRN gene was also found in zebrafish, Fugu, rat, mouse, cow and human [67] and was shown to have an overlapping pattern of expression in the central nervous system, similar to that of PrP^C^ [68,69]. In particular, the similarity between PrP and Sho is striking within the putative HC region, suggesting that Sho, but not Dpl, undergoes α-cleavage. 

As anticipated, Westaway’s group have shown that Sho is indeed cleaved at the putative α-cleavage site [68], although the sequence of the Sho-C1 has not yet been determined to identify the exact cleavage site. If Sho is cleaved to generate a “C1-like molecule”, it would be interesting to test whether expression of an “un-cleavable Sho” mutant can cause neuronal cell death like expression of the ΔF PrP mutant in the absence of wild-type Sho or PrP^C^. Obviously, Sho does not seem to compensate for the lack of PrP^C^, since ectopic expression of Dpl causes neuronal dysfunction in the absence of PrP^C^, but Sho should still be present, although Sho expression was not tested in the Rcm0, Ngsk, and Zrch II mice.

Collectively, the mammalian prion protein family consists of three members: ubiquitously expressed PrP^C^, testis-localized (although not exclusively) Dpl, and neuron-localized (although not exclusively) Sho. Since PrP^C^ null mice develop normally and are completely resistant to prion infections, another molecule has been suggested to compensate the function of PrP^C^. Sho is a good candidate for such compensation, but this does not seem to be the case. A recent publication by Westaway’s group has reported the analysis of mice deficient in both Sho and PrP^C^, and these mice were fertile and viable for up to 690 days of age [70]. These results directly contradict the hypothetical redundancy between PrP^C^ and Sho. In sharp contrast, however, Laude and Vilotte’s group [69] previously reported that *Sprn* knockdown in PrP^C^ deficient embryos using a lentiviral system resulted in embryonic lethality. The explanation for this discrepancy needs further investigation, but may be due to the different experimental strategies used.

## 5. The cellular compartment in which α-cleavage occurs

Prion infection into animals and humans results in the conversion of PrP^C^ into scrapie prion protein (PrP^Sc^) [71]. The conversion of PrP^C^ to PrP^Sc^ is clearly a posttranslational process [14]. One study proposed that the conversion process occurs intracellularly between the Golgi and the cell surface, since PrP^Sc^ was not released by phosphatidylinositol-specific phospholipase C (PIPLC) in the scrapie-infected murine neuroblastoma cell line, N2a [72]. In support of this mechanism, PrP^Sc^ was shown to be co-localized with the Golgi marker in murine N2a and Syrian hamster-derived HaB cells [73]. In contrast, Caughey’s group reported in 1991 that the precursor of PrP^Sc^ was eliminated from intact cells by treatments with PIPLC and trypsin, suggesting that the conversion of PrP^C^ to PrP^Sc^ occurs after the precursor reaches the cell surface in rat PC12 cells [74]. Collectively, it seems that the conversion occurs between the cell surface and the Golgi compartment and further study is warranted.

N2a is the most frequently and broadly used cell model for *in vitro* study of prion infection, although it is derived from murine cells, thereby limiting its use [75]. Many laboratories have reproducibly demonstrated prion infection into N2a cells, presumably due to a unique cellular attribute. The mechanism of PrP^C^ endocytosis has been studied frequently using N2a cells [76]. In 1993, Harris’s group used N2a cells to demonstrate that PrP^C^ cycles between the cell surface and an endocytic compartment [77]. In 2004, Kaneko’s group expressed fusion proteins of PrP with either EGFP or DsRed in N2a cells to show that both endogenous PrP^C^ and the fluorescent fusion proteins are localized at both the cell surface and the perinuclear compartment (PNC), part of which seems to be the Golgi [78]. Hachiya’s report also indicated that C1 is predominantly seen at the cell surface, while full-length PrP^C^ resides inside the cell, suggesting that full-length PrP^C^ can be recycled. The internalization of PrP^C^ in N2a cells is relatively rapid as demonstrated by Morris’s group, with more than 25% of the surface PrP^C^ internalized within 10 minutes [79]. In conclusion, evidence indicates that N2a cells exhibit proper intracellular trafficking of PrP^C^, which may be required for PrP^C^ to PrP^Sc^ conversion. 

In this scenario, prions (i.e. PrP^Sc^) may enter cells by binding to PrP^C^ on the cell surface and then PrP^C^ transports prions (i.e. PrP^Sc^) to the PNC, which seems to be either an endosomal compartment or the Golgi. Whether the massive conversion to prion occurs at the cell surface or in the PNC is unknown, but Tabrizi’s group reported strong detection of PrP^Sc^ at the plasma membrane and in the PNC in murine N2a cells, suggesting that both are candidate locations for conversion. In addition, they suggested that prion conversion occurs at the cell surface within 1 min, indicating that strong association of exogenous PrP^Sc^ with PrP^C^ occurs at the plasma membrane [80]. However, artificial GPI-anchorless PrP expressed in transgenic mice on a *Prnp*^0/0^ background was still converted to PrP^Sc^ after prion inoculation into the mice [12], indicating 1) that the conversion occurs in another compartment beyond the cell surface and 2) that there must be another endocytic pathway for PrP^Sc^ to enter cells. 

The α-cleavage of PrP^C^ may occur after expression on the surface, because both physiological and artificial GPI-anchorless PrPs are not cleaved [23]. Thus, if PrP^C^ is cleaved to generate C1, C1 does not take prions to the intracellular compartment, since it does not seem to be internalized. We have also found that PrP^C^ is rapidly internalized in N2a cells, but not in HpL3-4 cells, which are competent and incompetent for prion infection, respectively (data not shown), although other group has shown that PrP^C^ in HpL3-4 cells are rapidly internalized and HpL 3-4 cells are able to replicate prions [81]. HpL 3-4 cells that we obtained might have lost these phenotypes, suggesting that competent cells for prion propagation should have features of rapid PrP^C^ internalization and PrP^C^ accumulation in the Golgi. It would be interesting to check if other competent cell lines such as rat pheochromocytoma PC12, hypothalamic neuronal GT-1 cells, and the rabbit epithelial cell line RK13 [82] show similar endocytic features as N2a cells.

The identity of the cellular proteases responsible for α-cleavage seems to be important to address the location of α-cleavage event. In 1992 and 1995, Prusiner’s group suggested that a chymotryptic protease is responsible for the degradation of PrP^C^ to generate PrP^C^-II, because tosyl phenylalanyl chloromethyl ketone (TPCK), but not normal protease inhibitor cocktail, reduced the production of PrP^C^-II [19,20]. Alternatively, Prelli’s group reported in 1998 that the metal-chelating agents EDTA and EGTA show the highest activity to block α-cleavage of PrP^C^ [83], suggesting the involvement of metalloproteases, although the operative enzymes were not identified. In 2001, however, Checler’s group [84] provided the first evidence implicating the responsible metalloproteases as the disintegrins ADAM10 (A disintegrin and metalloprotease) and TACE (tumor necrosis factor a-converting enzyme or ADAM17) by using an *in vitro* cell culture system and a pharmacological approach, although they failed to completely block the generation of C1 and N1. In 2011, however, Glatzel’s group used neuron-specific *Adam* 10 knockout mice to show that ADAM10 does not have a direct role in the α-cleavage of PrP^C^, but rather it is responsible for the shedding of PrP^C^ from the surface of cell [85]. ADAM9 and ADAM10 are likely both involved in the shedding of PrP^C^ as supported by work from Hooper’s group [86]. Recently, Kong’s group demonstrated that ADAM8 is also a candidate for the α-cleavage of PrP^C^ [87]. Since mice with ADAM8 deficiency still exhibited residual C1 [87] and male and female *Adam8* -/- mice were viable and fertile [88], compensatory mechanism may be involved. Collectively, much work remains to clarify the exact enzyme mediating α-cleavage of PrP^C^. The best evidence currently available suggests that metalloproteases are involved. Interestingly, involvement of an enzyme responsible for the generation of PrP^Sc^ or C2, a pathogenic form of PrP^C^, has also been suggested [43]. This study reported that pharmacological inhibitors of calpains, caspases, and the proteasome, prevented the production of C2, while lysosomal proteases were not involved. Thus, C2 as well as C1 might be generated via physiological machinery. This needs further investigation because calpain-like activity is also suggested in normal processing of PrP^C^ [89].

## 6. Conclusions

Proteinse K (PK) treatment of brains infected with prions is the most convincing method to detect PrP^Sc^. The PK treatment gives rise to an N-terminally truncated form, the size of which is around Mr 27,000-30,000 (PrP 27-30). C1 is a completely different N-terminally truncated form, which is derived from normal cellular PrP^C^ and may have a physiological function. PrP27-30 is PK resistant and prion-associated, while C1 is PK sensitive and is the product of PrP^C^α-cleavage under normal condition. Recently several reports have shown the lack of detectable PK-resistant prion protein (PrPsen *vs*. PrPres) [90], but the infected host suffers from neurodegenerative dysfunction and the infectivity is transmissible [44,91]. These results demonstrate that pathogenic prion proteins are not always PK-resistant or detectable. The byproduct of the “α-cleavage phenomenon” is that PNGase F, which is able to remove almost all types of N-linked glycans, provides a valuable tool to detect both pathogenic PrP^Sc^ and PK-sensitive PrPsen. In some cases, PK treatment might not be necessary to detect abnormal prion proteins, because normal PrP^C^ shows only two bands, full-length PrP^C^ and C1, but brains of CJD patients show additional bands (i.e. C2) by PNGase F treatment alone [21]. 

It seems that there are at least two types of transgenic mouse models: “PrP^Sc^ type” and “ΔF type”. The “PrP^Sc^ type” mice generate PrP aggregation and neuropathology that is transmissible to another host, while “ΔF type” mice exhibit direct neurotoxicity, but it is counteracted by PrP^C^. Whether a mutation is associated with the “ΔF type” in humans is currently unknown. Alternatively, “ΔF type” mutations mimic neurotoxic signals caused by prions (PrP^Sc^ or PrPsen), since prions require endogenous PrP^C^ to induce CNS dysfunction.

The “α-cleavage phenomenon” provides an intriguing concept to improve our understanding of prion biology. When α-cleavage is blocked by mutations, it may induce either toxic ΔF type PrP or aggregated PrP such as PrP^Sc^. Thus, α-cleavage might be a protective mechanism to eliminate detrimental behavior of PrP^C^, since anchorless PrP is not cleaved and therefore tends to aggregate when overexpressed [52]. This may explain why endogenous expression of anchorless PrP^C^ is so minimal [23]. In addition, C1, a naturally occurring fragment of PrP^C^, has been shown to protect from prion disease by acting as a dominant negative inhibitor of PrP^Sc^ formation [92]. These reports enhance the importance of the “α-cleavage phenomenon”. Despite significant advances in recent years, substantially more research is necessary in the prion field. The interesting questions to be addressed in the future include: 1) What is the mechanism by which Dpl causes male sterility? 2) What is the normal function of Shadoo? 3) What enzymes are responsible to cleave PrP^C^ or Shadoo? 4) Where does conversion from PrP^C^ to PrP^Sc^ occur? 5) Where does α-cleavage occur? 6) What are the normal functions of N1 and C1? 7) What are the normal functions of anchorless PrP?
